# General consensus on multimodal functions and validation analysis of perinatal derivatives for regenerative medicine applications

**DOI:** 10.3389/fbioe.2022.961987

**Published:** 2022-10-03

**Authors:** Michela Pozzobon, Stefania D’Agostino, Maria G. Roubelakis, Anna Cargnoni, Roberto Gramignoli, Susanne Wolbank, Florelle Gindraux, Sveva Bollini, Halima Kerdjoudj, Mathilde Fenelon, Roberta Di Pietro, Mariangela Basile, Veronika Borutinskaitė, Roberta Piva, Andreina Schoeberlein, Guenther Eissner, Bernd Giebel, Peter Ponsaerts

**Affiliations:** ^1^ Department of Women’s and Children’s Health, University of Padova, Padova, Italy; ^2^ Laboratory of Biology, Medical School of Athens, National and Kapodistrian University of Athens, Athens, Greece; ^3^ Centro di Ricerca E. Menni, Fondazione Poliambulanza Istituto Ospedaliero, Brescia, Italy; ^4^ Department of Laboratory Medicine, Division of Pathology, Karolinska Institutet, Stockholm, Sweden; ^5^ Ludwig Boltzmann Institute for Experimental and Clinical Traumatology, The Research Center in Cooperation with AUVA Trauma Research Center, Austrian Cluster for Tissue Regeneration, Vienna, Austria; ^6^ Service de Chirurgie Orthopédique, Traumatologique et plastique, CHU Besançon, Laboratoire de Nanomédecine, Imagerie, Thérapeutique EA 4662, University Bourgogne Franche-Comté, Besançon, France; ^7^ Department of Experimental Medicine (DIMES), School of Medical and Pharmaceutical Sciences, University of Genova, Genova, Italy; ^8^ University of Reims Champagne Ardenne, EA 4691 BIOS “Biomatériaux et Inflammation en Site Osseux”, UFR d’Odontologie, Reims, France; ^9^ University of Bordeaux, INSERM, BIOTIS, U1026, Bordeaux, France; ^10^ Department of Medicine and Ageing Sciences, Section of Biomorphology, G. d'Annunzio University of Chieti-Pescara, Chieti, Italy; ^11^ Department of Molecular Cell Biology, Institute of Biochemistry, Vilnius University, Vilnius, Lithuania; ^12^ Department of Neuroscience and Rehabilitation, University of Ferrara, Ferrara, Italy; ^13^ Department of Obstetrics and Feto-maternal Medicine, Inselspital, Bern University Hospital, Department for BioMedical Research (DBMR), University of Bern, Bern, Switzerland; ^14^ Systems Biology Ireland, School of Medicine, Conway Institute, University College Dublin, Dublin, Ireland; ^15^ Institute for Transfusion Medicine, University Hospital Essen, University of Duisburg-Essen, Essen, Germany; ^16^ Laboratory of Experimental Hematology, Vaccine and Infectious Disease Institute (Vaxinfectio), University of Antwerp, Antwerp, Belgium

**Keywords:** perinatal derivatives, amniotic membrane and fluid stem cells, extracellular vesicles, tissue regeneration, regenerative medicine

## Abstract

Perinatal tissues, such as placenta and umbilical cord contain a variety of somatic stem cell types, spanning from the largely used hematopoietic stem and progenitor cells to the most recently described broadly multipotent epithelial and stromal cells. As perinatal derivatives (PnD), several of these cell types and related products provide an interesting regenerative potential for a variety of diseases. Within COST SPRINT Action, we continue our review series, revising and summarizing the modalities of action and proposed medical approaches using PnD products: cells, secretome, extracellular vesicles, and decellularized tissues. Focusing on the brain, bone, skeletal muscle, heart, intestinal, liver, and lung pathologies, we discuss the importance of potency testing in validating PnD therapeutics, and critically evaluate the concept of PnD application in the field of tissue regeneration. Hereby we aim to shed light on the actual therapeutic properties of PnD, with an open eye for future clinical application. This review is part of a quadrinomial series on functional/potency assays for validation of PnD, spanning biological functions, such as immunomodulation, anti-microbial/anti-cancer, anti-inflammation, wound healing, angiogenesis, and regeneration.

## 1 Introduction

**FIGURE 1:**
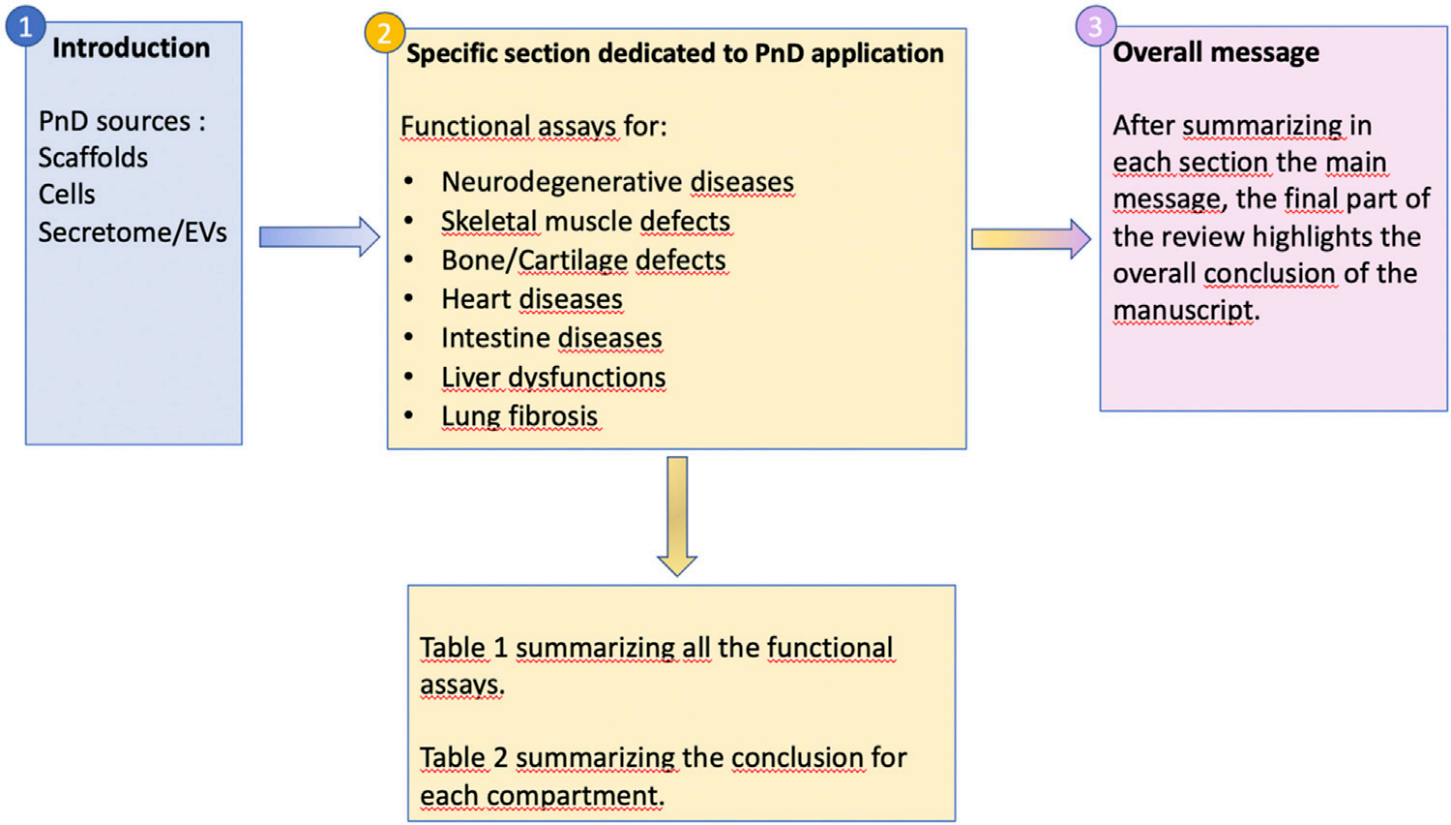
Flow chart of the manuscript organization.

Stem and progenitor cells, including their cellular and acellular derivatives, are increasingly applied as therapeutic agents for a variety of pathologies, not only in preclinical animal studies but—more importantly—in human clinical trials also. However, the field of regenerative medicine for non-hematopoietic diseases has evolved over the past 20 years from direct cell replacement to trophic support exerted by administered cell populations or their derivatives in order to induce tissue repair (i.e., improve the functionality of damaged/diseased cells/tissues) and/or regeneration (i.e., stimulate the growth of new cells replacing diseased cells/tissues). Nevertheless, many questions remain regarding the type of stem cell (product) needed, the minimal criteria such a product needs to comply with, and appropriate guidelines for determining the success of an applied therapeutic intervention, both in preclinical animal studies and human clinical trials. While this field has been dominated by studies using tissue and cell products obtained from adult stem cell sources, and besides pluripotent stem cell derivatives (embryonic stem/induced pluripotent stem cells, ES/iPSC), emerging alternatives can be found in perinatal tissues. Due to their naïvity and easy access, perinatal tissues and cells collected during pregnancy (AFS) and at full-term pregnancy, here referred to as Perinatal Derivatives (PnD), may provide important advantages over adult stem cell products, especially for non-hematopoietic (stem) cell products. The main non-hematopoietic PnD currently used in pre-clinical and clinical applications are amniotic fluid/tissue and cells isolated from the placenta, including the amniotic membrane and the umbilical cord. For a detailed description and correct nomenclature of the different perinatal cell types of therapeutic interest, we refer to a recent SPRINT-COST supported review manuscript by Silini and colleagues, in which a proposed consensus nomenclature for human perinatal tissues and cells has been published (Silini et al., 2020). Here we will further elaborate on the most frequently studied PnD, their phenotypic characteristics, and suggested therapeutic efficacy in different pathologies, including various tools to determine their potency, by means of functional assays and before (and after) administration. A general overview of the manuscript organization is provided in Figure 1.

**FIGURE 2:**
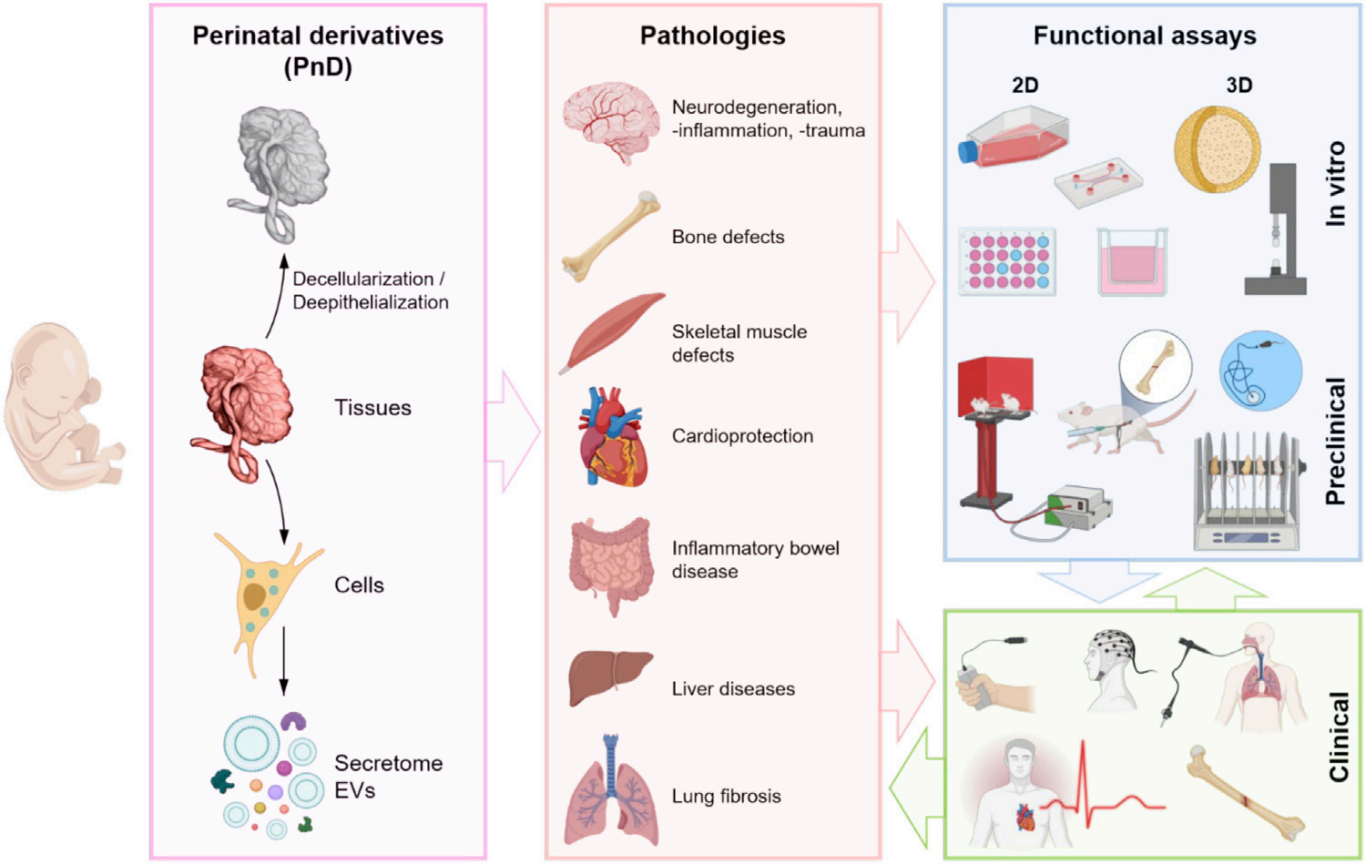
Schematic representation of the use of different PnD (tissues, cells, and paracrine derivatives—secretome (whole soluble factors including EVs) and isolated EVs), their application in different diseased organs and the functional assays. Created with BioRender.com.

More specifically, with this manuscript, a team composed of experts and pioneers in using PnD products in clinical and veterinarian treatments, afferent to the COST SPRINT Action (CA17116), aimed to offer a guide on when and how it may be advantageous to the employment of placenta-derived cells or scaffolds. We will consider the applications of different sources of PnD with a focus on the functional/potency assays for the regeneration of different affected organs (Figure 2).

Based on the unique ontogenetical and biological properties that characterize perinatal cells, different cells, spanning from infiltrating (maternal) immune cells to endothelial, epithelial, or mesenchymal stromal cells (MSC), have been isolated and studied during the last decades (Silini et al., 2020). These multipotent stem cells have been offering new, promising experimental applications for regenerative medicine and tissue engineering approaches. With its immunologically-privileged status (Zhang et al., 2017a), the PnD lends credence to its use as an allograft scaffold in tissue regenerative medicine. However, to widen a safe application, different strategies of nuclei depletion (decellularization or de-epithelization process) have been assessed to give rise to decellularized PnD (Gilbert et al., 2006; Crapo et al., 2011). After checking the quality of the decellularization process following standardized procedures of nuclei and DNA quantification (Gilbert et al., 2006; Crapo et al., 2011), naturally derived scaffolds, as from PnD, are thought to be an ideal and safe system to deliver chemokines and growth factors, to provide adequate structural and biomechanical microarchitecture in the damaged microenvironment (Jadalannagari et al., 2017; Dubus et al., 2022), with attention to avoid foreign body reaction and formation of the fibrous capsule (Keane et al., 2012; Hussein et al., 2016).

The development of a validation system designed according to international reference standards is an ambitious goal whose achievement still requires considerable effort toward the 1) cell-based strategies and 2) the cell-free strategies. Regarding the first, the efficacy of decellularized PnD (dP) combined with cells requires the employment of a conventional functional assay aimed at measuring the biochemical and mechanical properties of the dP on cell growth, differentiation, and functionality. In this scenario, the dP may act both as 1) a stem/progenitor-cell-like niche with key elements to control the regulation of stem cell fate and function, or 2) a platform for mature cells with the signals needed to initiate and maintain the differentiation of tissue-specific lineages and to support the cell repopulation after implantation. This approach relies on the ability of dP to promote the growth of cells with the enhanced potential to regenerate tissue damaged by injury, disease, or aging. This is the case of decellularized amniotic membrane (AM) and ultra-thin AM (Zhang et al., 2016) or Wharton’s jelly (Penolazzi et al., 2020; Dubus et al., 2022; Penolazzi et al., 2022) which have been produced as a suitable scaffold for the *ex vivo* expansion and delivery of stem and progenitor cells.

Regarding the cell-free strategies, the use of *in vivo* biological assays will allow testing the efficacy of dP extracellular matrix (dP-ECM) as a cell-free scaffold to produce a good regeneration guide.

A wide variety of clinical applications, after biocompatibility evaluation by subcutaneous implantation tests according to ISO/EN 10993 part 6 guidelines, have been reported for human amniotic membrane (hAM) / human amnio-chorionic membrane (hACM (Fenelon et al., 2019; Fénelon et al., 2021). *In vitro* cytocompatibility was assessed by extract cytotoxicity assay and contact cytotoxic assay according to the ISO/EN 10993 part 5 guideline.

Multicellular potency has been found critical in preclinical/clinical transplantation as much as PnD paracrine effects, subsidiary to support engraftment and long-term survival for donor cells.

It has been largely described as dP or perinatal cells have constitutive angiogenic and anti-inflammatory properties (C hoi et al., 2012) carried out by release mediators such as transforming growth factor beta 1 (TGF- β1), basic fibroblast growth factor (bFGF), epidermal growth factor (EGF), platelet-derived growth factor (PDGF), insulin-like growth factor 1 (IGF-1), vascular endothelial growth factor (VEGF), anti-inflammatory cytokines (i.e. interleukin (IL)-10), antimicrobial peptides (β-defensins, elafin), and tissue inhibitor of metalloproteinases (TIMP-1, 2, 3 and 4) (Hao et al., 2000; Mao et al., 2017a).

A remarkable property of natural scaffolds and more consistent in progenitor/stem cells is to secrete extracellular vesicles (EVs) commonly distinguished as: 1) macrovesicles (MVs) and 2) exosomes (Rani et al., 2015) vehiculating immunomodulatory or anti-inflammatory molecules in the form of miRNA, mRNA, proteins characteristic of cells of origin (Chen et al., 2010; Rani et al., 2015). Moreover, EVs may have a superior safety profile as compared to the cells they derived from since they do not replicate (Reis et al., 2016) nor cause microvascular embolism (Xiong et al., 2017) and can be stored without losing their properties (Wen et al., 2016). Thus, extensive research is currently underway to establish the potential use of mesenchymal stromal cells extracellular vesicles (MSC-EVs) for cell-free therapeutic applications in diseases, such as cardiovascular diseases (Fleury et al., 2014), liver failure (Maji et al., 2017), and respiratory diseases (Abreu et al., 2016). In addition, animal model-based studies suggest that exosome-sized or small EVs (sEVs), compared to their parental cells, may represent a novel cell-free medicinal product that is effective and can be safely stored without loss of function, as an alternative to cell-based therapies (El Andaloussi et al., 2013; Masyuk et al., 2013).

The role of PnD has been considered paramount in specific environments where support or corrective effects are needed, such as neurodegenerative diseases, skeletal muscle and bone repair, cardioprotection, intestine inflammation, and liver and lung chronic or congenital disorders. The use of either tissue patches, depleted from cells (decellularized hAM or umbilical cord), or perinatal cells/EVs have been validated in preclinical models and translated to the clinic (Figure 2). During the past year, our team composed of international experts on PnD technologies revised and compared existing standardized procedures validated to produce PnD medical products. The present work compiles minimum requirements and provides an overview of the minimal measures needed to translate PnD therapies to the clinic. Being supportive, Table 1 summarizes the applications of functional/potency assays of PnD in research and the clinic (also see Table 1 for more details).

**TABLE 1:** Overview of the functional assays for the different compartments analyzed in the present manuscript.

Source of PnD	Functional assay			Pathology/model	References
	*In vitro*	*In vivo*/preclinic	Clinic		
hUC-WJ-MSC from term or preterm birth	Proliferation capacity			Spinal cord injury, Parkinson’s disease, Alzheimer’s disease, Perinatal brain injury	Petrenko et al. (2020), Oppliger et al. (2017)
	Protein composition of secretome Iinflammatory/anti-inflammatory factors Adhesion molecules				
	Neuroprotective effect on oxidative stress				
	Stimulation of neurite outgrowth				
	Metabolic activity under serum starvation Expression of functional genes				
	Glial differentiation of neural progenitors				
	Oligodendrocyte lineage markers gene expression				
hUC-WJ-MSC-EVs	Immunocytochemistry and expression of oligodendrocyte lineage markers			Perinatal brain injury	Joerger-Messerli et al. (2021), Thomi et al. (2019)
	miRNA cargo analysis				
	Inflammatory-related gene and protein expression and cytokine secretion				
	sEV uptake: confocal microscopy, flow cytometry				
Amnion muscle combined graft (AMCG)		Grasping tests. Scar formation toluidine blue staining		Rat’s median nerve defect	Marchesini et al. (2018)
hAM			Muscle Testing of Lister	Loss of substance of the median nerve	Riccio et al. (2014)
			Jamar test		
			Classification of Sakellarides		
			Quick-DASH evaluation questionnaire		
hAM+ hUC-WJ-MSC			Modified Medical Research Council classification (MRCC)	Radial nerve injury	Li et al. (2013)
			Electromyogram (EMG)		
hAEC		Stress relaxation tests		Injured brachial plexus nerve in rabbit	Jin et al. (2015)
Decellularized WJ	DNA quantification	Direct and indirect cytotoxicity evaluation after subcutaneous implantation (ISO/EN 10993 part 5-6 guidelines)		Bone/Cartilage regeneration	Hussein et al. (2016)
	DAPI staining and visualization				
	Histological staining				
	Growth factor release				
	MALDI TOFF proteomic assay				
hAM	Silver nitrate, Von Kossa and Alizarin red stainings	2D or 3D-Radiography		*In vitro* and *in vivo* osteogenic potential	Lindenmair et al. (2010), Gualdi et al. (2019), Fenelon et al. (2021), F enelon et al. (2020)
	Immunohistochemistry for osteogenesis	Histology Immunohistochemistry to evaluate bone formation			
	Energy dispersive X-ray (EDX)				
	X-ray diffraction (XRD)				
	Metabolic activity assay				
hUC-WJ-MSC + muscle scaffold	Histological staining for quantification of fibrous area	Evaluation of maximal isometric contractile force		Volumetric muscle loss	Qiu et al. (2018), Hadipour et al. (2021); Izadi et al. (2021), Magarotto et al. (2021)
hUC-WJ-MSC-EVs + muscle scaffold	Quantification of centrally located myofibers or cross section area				
Decellularized hAM	Immunofluorescence staining for regenerating muscle, nerve, blood vessels markers				
	Immunofluorescence and flow cytometry for M2 macrophage polarization				
hAM	Live imaging and cell tracking to study cell motility			Muscle injury (3D model)	Hasmad et al. (2018), Hadipour et al. (2021)
	Measuring of fibers angle for alignment detection				
hUC-MSC	Histological staining for quantification of fibrous area	Gait/walk analysis		Chemical muscle damage	Su et al. (2019), Arutyunyan et al. (2019)
	Hystomorophometric analysis			Unilateral hindlimb ischemia	
	Immunofluorescence staining for regenerating blood vessels				
	Immunofluorescence and cytometry for M2 macrophage polarization				
hUC-WJ-MSC,	Immunofluorescence staining for regenerating muscle markers	Grip analysis		Duchenne Muscular Dystrophy	Mishra et al. (2020), Park et al. (2021), Bier et al. (2018)
hUC-WJ-MSC-EVs	Histological staining for quantification of fibrous area				
	Transcriptome analysis for myogenic markers or miRNA				
hUC-WJ-MSC	Immunofluorescence staining for regenerating muscle markers			Sarcopenia	Wang et al. (2018)
	Apoptosis detection				
hUC-WJ-MSC-EVs	Immunofluorescence staining for regenerating nerve markers	Gait/walk analysis		Sciatic nerve resection	Ma et al. (2019)
	Hystomorophometric analysis				
hAFSC-CM	Cell viability assay			Myocardial ischemic injury	Balbi et al. (2019a)
	Assessment of Ca2+ transients				
	Capillary network formation assay				
hAFSC-CM	*Ex-vivo* histological evaluation of fibrosis and scarring on murine myocardial tissue sections	*In vivo* cardiac function analysis by ultrasounds		Myocardial ischemic injury	Balbi et al. (2019b)
hAFSC-EVs	*Ex-vivo* Immunofluorescence for inflammatory cells (MPO) and TUNEL assay for cardiomyocyte apoptosis (cardiac troponin I)				
	*Ex-vivo* Immunofluorescence for neoangiogenesis and activation of epicardial progenitor cells				
hAFSC-EVs	Cell viability assay			Myocardial ischemic injury	Takov et al. (2020)
	Modified Boyden's chamber assay on HUVEC				
	WB analysis of PI3K signaling				
	Proteome profiler human phospho-kinase array on HUVEC				
	*Ex-vivo* TTC staining of tissue sections				
hAFSC-CM	Cell viability assay	*In vitro* blocking experiments using anti-Il6 and anti-Cxcl1 antibodies and PI3K inhibitor on murine neonatal cardiomyocytes exposed to doxorubicin		Mouse neonatal cardiomyocytes or murine cardiac tissues exposed to doxorubicin. Doxorubicin-induced cardiotoxicity	Lazzarini et al. (2016)
	Immunocytochemistry for prosenescent and apoptotic marker	*In vivo* cardiac function analysis by ultrasounds			
	Immunostaining for DNA damage				
	RNA microarray				
	Real time qRT-PCR				
	WB analysis				
	MitoTracker Deep Red staining				
	Oxidative phosphorylation activity				
	*Ex-vivo* biochemical evaluation of mithocondrial metabolism				
hUC-MSC	Cell viability, TUNEL and ApopTag assay	*In vivo* cardiac function analysis by ultrasounds		Myocardial ischemia/reperfusion injury	Danieli et al. (2015), Santos Nascimento et al. (2014)
hAMSC-CM	WB analysis				
	Boyden chamber assay on endothelial progenitors				
	Angiogenic assay on Matrigel on endothelial progenitor cells				
	*Ex-vivo* TTC staining				
	*Ex-vivo* histological analysis of fibrosis and infarct size				
	Ex-vivo immunostaining for angiogenesis				
hUC-MSC-EVs	*Ex-vivo* real time qRT-PCR	*In vivo* cardiac function analysis by ultrasounds		Myocardial ischemic injury	Huang et al. (2020)
	Ex-vivo ELISA	*In vitro* functional evaluation of mechanism of action by SOX6 knockdown and miR-19 inhibition			
	*Ex-vivo* histological evaluation of myocardial injury and cell apoptosis				
	*Ex-vivo* TTC staining				
	Cell viability assay				
	Evaluation of ROS production				
	WB analysis				
hUC-MSC		Evaluation of weight loss, intestinal mucosal injury, colon shortening, and reduced clinical disease phenotype.	Decreased IBD activity index, enhance healing process	DSS murine model. Fistula	Banerjee et al. (2015), Panhua et al. (2019), Wang et al. (2020), Hossein-khannazer et al. (2021)
		Evaluation of intestinal tight junctions, autophagy markers and VEGF signal at the injured site			
hUC-MSC-EVs		Evaluation of restored mucosal barrier repair with inteleukin and TNFa signaling analysis		DSS murine model	Mao et al. (2017b), Tolomeo et al. (2021), Yang et al. (2021)
MSC isolated from AF and chorionic plate		Detection of anti-inflammatory interleukins induced liver recovery.		CCl₄-injured NOD/SCID mice	Zagoura et al. (2012), Peng et al. (2014)
MSC isolated from chorionic plate		Evaluation of the expression levels of α-smooth muscle actin (α-SMA) and Col I		CCl₄-injured rat	Lee et al. (2010)
hAEC	Evaluation of CYP3A4 activity and inducibility, albumin secretion, ammonia metabolism, the ability to efflux rhodamine or store bile acids, lipids or glycogen. Production of multicellular organoids with hepatic function in coculture with HUVEC and MSC			Liver organoid. *In vitro* 3D culture model of hepatocytes	Furuya et al. (2019), Pettinato et al. (2019), Ramachandran et al. (2015)
hAM stromal extract	Western Blot analysis. Detection of proliferating cells			*In vitro* model of myofibroblasts culture	Li et al. (2008)
hAEC	Proliferation assay. Immunostaining for the assessment of transformation of fibroblasts to myofibroblasts. Colorimetric assay for collagen. Gene expressions of TGF-β, PDGF-α, and PDGF-β by real-time PCR. Matrix Metalloproteinase (MMP-2 and MMP-9) Activity Assay in culture supernatants (by gelatin zymography)	Hystomorophometric analysis. Quantification of collagen. Immunofluorescence lung area fraction occupied by CD45-positive cells, macrophages and neutrophils. Gene expression. Pro-inflammatory cytokines analysis. Protein zymography		*In vitro* model of primary mouse lung fibroblasts culture. Bleomycin-induced lung fibrosis model	Vosdoganes et al. (2013), Murphy et al. (2011), Vosdoganes et al. (2013), Tan et al. (2014), Tan et al. (2015), Moodley et al. (2010)
hAEC-CM hAEC-Exo	Chemotactic Macrophage Migration Assay. Colorimetric assay for collagen. Immunofluorescence staining			*In vitro* model of primary mouse lung macrophages culture. *In vitro* model of human lung fibroblasts culture/ bleomycin-induced lung fibrosis model	Murphy et al. (2012)
hAMSC		Hystomorophometric analysis. Immunostaining and gene expression of inflammatory mediators and ECM proteins. Flow-cytometry of bronchoalveolar lavage		Bleomycin-induced lung fibrosis model	Carg noni et al. (2020), Moodley et al. (2013)
hAMSC-CM		Lung expression of inflammatory mediators by Cytometric Bead Array. Lung PGE2 levels by EIA assay		Bleomycin-induced lung fibrosis model	Cargnoni et al. (2014)
Human Fetal Membrane-Derived Cell (hAEC+hAMSC+hCMSC)		Hagood's score. Collagen deposition analysis. Hystology on inflammatory cell infiltrations		Bleomycin-induced lung fibrosis model	Cargnoni et al. (2009)
hUC-MSC		Histopathological analysis. Ashcroft's score. Colorimetric quantification of collagen. Gene expression by quantitative real-time PCR. Immunofluorescence staining	28-day mortality. Clinical symptom improvement Hematologic indicators Lung imaging changes. Length of hospitalization PaO2/FiO2 ratio. Dynamics of cytokines, and IgG and IgM anti-SARS-CoV-2 antibodies	Bleomycin-induced lung fibrosis model. Severe COVID-19 moderate and severe COVID-19. Acute respiratory distress syndrome (ARDS) in COVID-19	Moroncini et al. (2018), Shu et al. (2020), Lanzoni et al. (2021)

Neurodegeneration, -inflammation, -trauma; Bone defects/cartilage; Skeletal muscle defects; Heart diseases; Inflammatory bowel disease; Liver disease; Lung fibrosis.

Crucial aspects of qualification and functional characterization of the final products will be described and discussed. Within the next paragraphs, we aim to bring to light the standardized procedures used to produce a specific regenerative effect. Table 2 provides an overall conclusion for each of the compartments discussed below.

**TABLE 2:** Conclusion at a glance.

Conclusion at a glance for each analyzed compartment	
Neuronal	*in vitro* assays are not uniformly used to evaluate novel therapeutic strategies for CNS disorders, limiting uniform conclusions.
	3D models and organoids for *in vitro* CNS research may be a major step forward towards clinically meaningful readouts
Muscle	*in vitro* analysis on paracrine factors released by MSC have been proposed as executive of the mode of action for PnDs in skeletal muscle repair.
	pleiotropic effects can be validated *in vivo*.
Bone	*in vitro* functional assays identified activities such as immunomodulation, proangiogenic and trophic factor release after employment of AM
Heart	*in vitro* assays describe cardioprotective effects.
	*in vivo* validation of PnD therapeutic efficacy (by assessing cardiac function via ultrasound analysis)
Intestine	*in vivo* animal models to validate PnD products is a prospective direction to modulate microbial structure or intestinal homeostasis.
Liver	transcriptomic analysis coupled with metabolic activity are required to validate progenitor/stem cell maturation into functional hepatocytes.
	3D PnD-derived organoids are innovative, instrumental tools to study angiogenesis, innervation and to improve survival of encapsulated hepatocytes
Lung.	limitations of the *in vitro* 3D lung models to recapitulate all disease features
	*in vivo* models evidenced the anti-inflammatory and anti-fibrotic actions of PnD (cells, secretome and EVs) encouraging application in clinical studies

## 2 Specific sections dedicated to PnD applications

### 2.1 Role of perinatal derivatives in treatment of neurodegenerative diseases

Novel treatment suggestions for neurodegenerative disorders applying cell or cell-derived products, including those from perinatal sources, primarily rely on the beneficial paracrine effects of the applied products. Although PnD have been shown to display trans-differentiation capacity into neural-like cells *in vitro*, or shown to home to the regions of interest upon transplantation, their differentiation capacity into neural cell types and the replacement of damaged neural cells *in vivo* following grafting plays only a minor (if after all) contribution to the beneficial effect PnD can display as a treatment modality for neurodegenerative disorders. More likely, as neurodegeneration is often accompanied by out-of-balance neuroinflammation, PnD interventions aid in resolving chronic inflammatory processes, induction of neuroprotection by the release of neurotrophic factors, and by stimulation of endogenous neurogenesis, with all three contributing to a successful neuro-regenerative potential (Volkman and Offen, 2017).

#### 2.1.1 Functional assays to demonstrate a neuroprotective effect of perinatal derivatives on neural stem/progenitor cells and neuronal cells after oxidative stress

Oxidative stress following acute or chronic hypoxic-ischemic insults, such as ischemic stroke, hemorrhagic stroke, or hypoxic-ischemic encephalopathy, is a common hallmark of many neurodegenerative diseases. A number of assays have been developed to assess the potential of PnD to reduce or resolve stress responses during/after an ischemic insult. Hereto, neural stem (NSC) or progenitor cells (NPC), neuronal primary cells, or cell lines (such as the mouse neuroblastoma cell line Neuro 2a) are exposed to conditions mimicking a hypoxic-ischemic insult, such as oxygen-glucose deprivation (OGD), possibly followed by reoxygenation or the exposure to H_2_O_2_ (Petrenko et al., 2020). Different types of PnD may then be added in direct or in indirect co-culture (transwell culture system) with the neural cells. Alternatively, the neural cells’ culture medium can be supplemented or replaced with a conditioned medium containing the PnD’s secretome (fractions). Since NSC/NPC have the potential to differentiate into all neural cell types, the effects of the PnD treatment on the cell fate specification and differentiation can be assessed by measuring the expression of cell type-specific markers along with the documentation of nuclear and cytoplasmic morphological changes in cells undergoing differentiation. With neuronal cell death being a hallmark of hypoxic-ischemic insults, in the first instance changes in neuronal apoptotic and necrotic outcome measures, such as standard viability assays as well as DNA fragmentation or the expression of molecules associated with apoptotic pathways (e.g., caspases), are valid indicators of the neuroprotective potential of PnD. Depending on the disease model, more specific assays such as the effect of PnD on neurite outgrowth (measured as the total area of neurites) of dorsal root ganglion (Petrenko et al., 2020) or other neurons will complement these outcome measures.

#### 2.1.2 Functional assays to demonstrate a pro-myelination effect of perinatal derivatives on oligodendrocyte (progenitor) cells

Myelination of neuronal axons is a prerequisite for the efficient transmission of electrical signals in the central nervous system (CNS). Many neurodevelopmental or degenerative disorders disrupt myelination or myelin homeostasis. Oligodendrocytes in the CNS and Schwann cells in the peripheral nervous system (PNS) are the cells that build the myelin sheath enwrapping the axons. Oligodendrocytes differentiate from NSC/NPC into oligodendrocyte progenitor cells (OPC), differentiation-committed precursors, newly formed oligodendrocytes, myelin-forming oligodendrocytes, and finally mature oligodendrocytes. This oligodendrocyte fate specification is used to model the potential of PnD to support processes involved in myelination. Hereto, NSC/NPC are directly or indirectly co-cultured with PnD or the culture medium supplemented with a conditioned medium derived from PnD cultures. Differentiation into the different stages of oligodendrocyte development is observed by morphological changes and the expression of stage-specific proteins such as chondroitin sulfate proteoglycan 4 (CSPG4) and platelet-derived growth factor receptor alpha (PDGFRA), markers of oligodendrocyte progenitor cells, galactosylceramidase (GALC), a marker of immature oligodendrocytes, or the mature oligodendroglial marker myelin basic protein (MBP) (Oppliger et al., 2017). Improvement of damage or developmental arrest can also be assessed by co-culturing PnD or administering their secretome to oligodendrocyte cell lines (e.g., MO3.13, (Joerger-Messerli et al., 2021)) or primary OPC after lipopolysaccharide (LPS)- and/or OGD-mediated injury.

#### 2.1.3 Functional assays to demonstrate anti-inflammatory effects of perinatal derivatives on microglia and astrocytes

Microglia are the resident immune cells of the CNS and act as scavengers to remove damaged cells or pathogens. Under pathological conditions, microglia react to danger or pathogen-associated molecular patterns (DAMP/PAMP), proliferate, migrate, undergo morphological changes, and secrete inflammatory molecules such as cytokines, chemokines, and neurotoxic factors. This stimulation can be mimicked *in vitro* by the exposure of primary microglia (Stansley et al., 2012), mixed glial cells (Thomi et al., 2019), immortalized (Stansley et al., 2012; Thomi et al., 2019; Spinelli et al., 2020), or iPSC-derived (Hasselmann and Blurton-Jones, 2020) microglial cell lines to LPS or a combination of interferon-gamma and tumor necrosis factor-alpha (IFNγ+TNFα) (Lively and Schlichter, 2018), with or without hypoxia. Indirect co-cultures with the microglial cells in the bottom and the PnD in transwells, or the addition of PnD-conditioned medium to microglial cells are used to examine the PnD immunoregulatory effects on microglial activation by measuring microglial proliferation, the expression or secretion of inflammatory molecules and by documenting morphological changes (Cao et al., 2019). Alternatively, organotypic brain explant models allow for stimulating glial cells in their niche and the context of pathological cell interactions (Holloway et al., 2021). Furthermore, the recent development of neuro-immune competent organoid models may even shed further light on the diffusion properties of PnD-derived therapeutic actors within a brain-like environment.

Astrocyte activation is a second important hallmark in many neuroinflammatory and degenerative diseases. Primary astrocytes or glial cell cultures enriched in astrocytes (Gorina et al., 2009) can be activated by the treatment with LPS (Roboon et al., 2021) or by OGD (ischemia-reperfusion (Wu et al., 2021)) or H_2_O_2_ (Wen et al., 2021) in indirect (transwell) co-culture systems. Outcome measures include the production of reactive oxygen species (ROS), the upregulation of the intermediate filament proteins glial fibrillary acidic protein (GFAP) and vimentin, and the presence of complement C3^+^ astrocytes or reactive astrocyte-induced neuronal cell death (Liddelow et al., 2017).

#### 2.1.4 Functional assays to demonstrate the influence of perinatal derivatives on blood-brain barrier integrity

CNS injury or hypoxic-ischemic and inflammatory insults are accompanied by an increase in the blood-brain barrier (BBB) permeability, resulting in the extravasation of proteins and uncontrolled immune cell trafficking (Cecchelli et al., 2014). Advanced *in vitro* BBB models include brain microvessel endothelial cells, pericytes, and astrocytes (Jamieson et al., 2019) grown in transwell cell cultures or microfluidic devices (Campisi et al., 2018). Small molecule tracers, such as the fluorescent FITC-dextran (Campisi et al., 2018), are added into the upper chamber or outer compartment and are used to monitor the permeability of the BBB model upon stimulation with inflammatory molecules such as TNFα (Chen et al., 2016). The influence on BBB permeability by PnD secretomes can be tested by adding a PnD-conditioned medium to the culture system. Additionally, readout systems include the expression of adhesion molecules and tight junction proteins on the endothelial cells that are involved in the rolling, tethering, and extravasation of peripheral immune cells from the bloodstream to the brain. Transendothelial electric resistance (TEER) is used to measure the integrity of tight junctions in transwell BBB culture models (Srinivasan et al., 2015).

#### 2.1.5 Functional assays to demonstrate the influence of perinatal derivatives on peripheral nerve repair

hAM has been evaluated as nerve wraps or conduits for the repair of peripheral nerve injuries *in vitro* in human and pre-clinical models (Wolfe et al., 2022) and humans (Fénelon et al., 20212021). hAM alone or associated with adult or perinatal cells (Fénelon et al., 2021), such as allogeneic hUC-MSC (Li et al., 2013), has been evaluated. In animal models, allogeneic hUC-MSC alone or coupled to additional scaffolds like PCL polycaprolactone (PCL) or chitosan were delivered at the site of injury (Bojanic et al., 2020), supplemented with systemic erythropoietin injection (Ülger et al., 2021).

In addition, human amniotic membrane epithelial cells (hAEC) (Jin et al., 2015), hAMSC (Chen et al., 2022), hAF stem cells (Su et al., 2018), or hUC-MSC (Bojanic et al., 2020) have been tested alone or coupled to additional scaffolds like PCL or chitosan or embedded in fibrin hydrogel-containing polylactate (PLA) nerve conduits (Su et al., 2018). For sciatic nerve injury models, Schwann cell proliferation and migration were used as *in vitro* assays (Chen et al., 2022). Non-invasive, conventional magnetic resonance imaging (MRI) coupled to a diffusion tensor MRI (DTI)-based fiber tractography assay has been used in the minipig model to follow the repair of sciatic nerve injury after hAF stem cell grafting (Su et al., 2018). Stress relaxation and creep testing were performed to assess the viscoelastic behavior of strained brachial plexus samples, and grooming behavior was scored as an indicator of forepaw motor function in a rabbit model (Jin et al., 2015).

Animal studies and clinical trials have highlighted their role in preventing the recurrence of perineural adhesions, reducing extra- and intra-neural fibrosis, accelerating nerve repair/regeneration, and improving nerve function (Lemke et al., 2018; Bour geois et al., 2019; Fénelon et al., 2021; Mirzayan et al., 2021). Different types of nerve injuries, including those affecting lower or upper extremity and thorax were assessed for: gap, transection, neurotmesis, crush, epineurectomy, induction of chemical fibrosis, and partial transection.

Histological (HE, Toluidine Blue, Oil Red O staining) or immunohistological analysis have allowed qualitative/stereological analysis of the gross morphology or microstructure of: **1)** nerve parameters such as axon regeneration—increase of axon size—number—diameter—density, axonal migration, myelin sheath thickness or nerve fiber diameter; **2)** muscle mass innervated by the transected then repaired nerve and **3)** muscle atrophy attenuation (Bour geois et al., 2019; Bojanic et al., 2020; Ülger et al., 2021; Wolfe et al., 2022). Retrograde axonal transport was estimated through fluorogold-labeled neuron counts after hUC-MSC grafting (Bojanic et al., 2020).

Neuromorphometry employs transmission electron microscopy or high-resolution light microscopy in which the number of myelinated fibers, the total number of nerve fibers, axon diameter, myelin thickness, G-ratio, and N-ratio were the most commonly reported outcomes (Marchesini et al., 2018; Wolfe et al., 2022).

In a rat scarring model for recurrent adhesions in the peripheral sciatic nerve, a vital hAM has been demonstrated to prevent adhesion, improve the level of extra- and intra-neural fibrosis, and support nerve regeneration, and also reflected in the sciatic functional index (Lemke et al., 2018).

Functional motor assessments included various types of static or dynamic gait analysis, strength measurements, or analysis of other specific behavior (Su et al., 2018; Bour geois et al., 2019; Ülger et al., 2021; Wolfe et al., 2022). The sciatic functional index (SFI) is an index of the functional condition of rat sciatic nerve based on measurements made from walking tracks (Ülger et al., 2021). Functional sensory outcomes measures also included withdrawal latency reflex (WRL) tests, tactile tests, or heat cutaneous allodynia (Wolfe et al., 2022).

Electrophysiological tests were performed in the pre-clinical model using a bipolar stimulating electrode placed under the nerve proximal to the hAM graft. Recordings were conducted using superficial electrodes and performed with an electromyogram (EMG). The peak amplitude of the compound muscle action potential (CMAP), CMAP latency of onset, and nerve conduction velocity (NCV) values were calculated (Su et al., 2018; Bour geois et al., 2019; Wolfe et al., 2022).

In humans, non-invasive ultrasound was used to determine neuroma formation and nerve regeneration determined based on EMG (Li et al., 2013).

After hAM grafting, sometimes associated with the hUC-MSC application, sensory and motor function restoration was evaluated based on the Modified Medical Research Council classification (MRCC), categorizing superficial cutaneous pain and tactile sensibility (Li et al., 2013). Additional tests include the Lister test for the intrinsic muscles of the hand, grip power and pinch strength measurements, and Jamar or Sakellarides tests (Riccio et al., 2014).

Finally, satisfaction of patients was assessed by means of the pain disappearance and the Quick Disabilities of the Arm, Shoulder, and Hand (Quick DASH) which evaluates 30 items that measure: 1) physical abilities (21 items); 2) severity of symptoms (5 items), and 3) social skills or roles (4 items) (Marchesini et al., 2018).

#### 2.1.6 Conclusion

The advantage of CNS *in vitro* cell culture systems, compared to animal models, is the relative ease of use, their potential for standardization, and the comparatively short interval from the models’ onset to the collection of data. Unfortunately, these assays either alone or in combination are not uniformly used throughout various studies evaluating novel therapeutic strategies for CNS disorders. Therefore, this field may majorly benefit from defining a standardized approach to select a number of appropriate elementary *in vitro* assays for which PnD should pass before proceeding to animal studies and/or human clinical trials. Furthermore, in line with the view of *in vitro* models contributing to the 3R principles in animal experimentation, the development of 3D models and organoids for *in vitro* CNS research may be a major step forward toward clinically meaningful readouts. Once established, this may increase the predictivity of therapeutic success of various PnD.

### 2.2 Role of perinatal derivatives in treatment of the skeletal-muscle injury

The most challenging aspect of skeletal muscle regeneration is the need for reconstruction of muscle fiber architecture in order to restore tensile force and function. With this in mind, various kinds of supportive scaffolds have been employed for tissue engineering purposes, to guarantee correct myoblast alignment and to induce their fusion. Several approaches already involved the use of scaffolds of natural origins like decellularized PnD to drive myofiber production in damaged muscle.

#### 2.2.1 Functional assays to demonstrate the influence of perinatal derivatives-derived scaffolds on skeletal muscle repair

Experimentally, decellularized scaffolds produced from human amniotic membrane have been proven effective in muscle repair and force regain when combined with training in a rat volumetric muscle loss (VML) model (Izadi et al., 2021). This approach furthermore led to a significant increase in the number of the newly formed centrally nucleated myofibers compared to untreated muscles, an important parameter often evaluated in such models because it is directly correlated to the fusion of myoblasts occurring during muscle regeneration (Wang et al., 2018; Hadipour et al., 2021; Izadi et al., 2021; Magarotto et al., 2021). Moreover, tissue regeneration after scaffold implantation was not only visible by histological methods, but at a functional level. Here electrophysiological methods allowed to demonstrate increased isometric contraction force of the muscle after nerve stimulation (Qiu et al., 2018; Izadi et al., 2021; Magarotto et al., 2021). In an alternative approach, the peculiar tissue architecture was reproduced in artificial implantable scaffolds with the addition of electrospun fibers of polymers like poly(lactic-co-glycolic acid) PLGA or PCL onto the decellularized amniotic membrane. This guaranteed optimal mechanical properties (strength and elasticity) and cell orientation, as evaluated by measuring the angle of the generated fibers or with live imaging techniques to track cell motility (Hasmad et al., 2018). Furthermore, stem cell differentiation was favored toward a myogenic phenotype, defined by the expression of regenerating muscle markers like MyoD, MyoG, Mhc (Hadipour et al., 2021; Izadi et al., 2021; Magarotto et al., 2021; Park et al., 2021).

#### 2.2.2 Functional assays to demonstrate the influence of perinatal derivatives on skeletal muscle repair

The employment of a scaffold is often not sufficient for complete regeneration in case of extensive tissue damage. In this situation, an effective approach is the administration of cell types that show myogenic potential (Mishra et al., 2020) or are active in the crosstalk with the other cell types that reside in the muscle niche. UC-MSC are active in muscle repair thanks to the interaction with the inflammatory compartment, which triggers and supports regenerative mechanisms. Importantly, they are not only involved in the production of myogenic miRNAs (Mishra et al., 2020; Sun and Karaoz, 2020; Park et al., 2021), but they are also capable to 1) promote macrophage polarization toward an M2 pro-regenerative phenotype, as characterized by the expression of both CD68 and CD206 (Qiu et al., 2018; Arutyunyan et al., 2019), and 2) reduce neutrophil-mediated acute inflammation, indicated by reduced infiltration of Ly6G^+^ cells at the site of injury (Su et al., 2019). Mechanistically, MSC were found to reduce chronic inflammation in sarcopenia through TNFα and IL-6 downregulation (reducing M1-oriented pro-inflammatory signals) and IL-4 and IL-10 upregulation (stimulating M2-oriented anti-inflammatory signals (Wang et al., 2018). For their immunomodulatory and antifibrotic properties, they were also proposed for the treatment of Duchenne’s Muscular Dystrophy (Sun and Karaoz, 2020; Park et al., 2021). Moving from cell-based interventions to acellular therapeutics, similar immunomodulatory and/or anti-fibrotic effects were also exerted by their secretome. The mechanism of action of MSC-EVs in muscle regeneration is still under investigation. However, evidence exists that they can modulate the inflammatory response and direct it toward regeneration with the reduction of fibrosis, a parameter quantified by the histological analysis of collagen deposition in the regenerating tissue (Bier et al., 2018; Magarotto et al., 2021). MSC-EVs also act on neuromuscular junctions, repairing axons and restoring nerve physiology (Ma et al., 2019; Magarotto et al., 2021), which is paramount for muscle function, evaluated *in vivo* by grip or gait analysis (Ma et al., 2019; Su et al., 2019).

#### 2.2.3 Conclusion

The multifaceted properties of PnD derivatives are attracting scientific interest for the development of new therapeutic applications in the regenerative medicine field of muscle regeneration. Besides the structural and mechanical support, it appears evident that the paracrine factors released by MSC such as the EVs are the executive of the mechanism of action of PnD in skeletal muscle repair. However, this complex aspect is still under investigation.

When skeletal tissues are damaged, natural scaffolds with their own growth factors and cytokines but also adjuvanted with MSC and EVs stimulate a favorable microenvironment. The promoted regeneration by the induction of pleiotropic effects such as, among others, immune-modulating and pro angiogenic can be validated *in vivo*. In this paragraph indeed, we underlined that *in vivo* functional assays are the best way to prove the PnD derivatives effects.

### 2.3 Role of perinatal derivatives for bone repair

Bone tissue engineering has emerged as an interdisciplinary strategy combining biomaterials, cells, and/or biologically active molecules, aiming to reconstruct injured or lost bone. PnD and in particular, perinatal tissues and derived cells have been used for that purpose. Orthopedic surgeons successfully apply cryopreserved or decellularized and lyophilized bone allografts. Thus, de-epithelialized or decellularization and/or lyophilization have been used accordingly for perinatal tissues to improve their storage, their biocompatibility, and their capacities to act as a scaffold.

#### 2.3.1 Functional assays to demonstrate the osteogenic potential of the amniotic membrane

Both human amniotic membrane MSC (AMSC) and hAECs display *in vitro* and *in vivo* osteogenic differentiation potential (Kmiecik et al., 2015). It was reported that under osteogenic conditions *in vitro*, intact hAM became mineralized and expressed markers of early and late osteoblastic differentiation, as demonstrated by a significant rise in calcium contents, induction of alkaline phosphatase (AP) activity, RUNX2, AP, BMP-2, and BMP-4 mRNA expression and osteocalcin staining colocalizing with von Kossa/alizarin red staining (Lindenmair et al., 2010).

Similarly, anti-collagen I immunohistochemistry was carried out on intact osteodifferentiated hAM to highlight osteoblastic differentiation potential (Gualdi et al., 2019). Furthermore, Energy dispersive X-ray (EDX) and X-ray diffraction (XRD) were used as a novel approach to qualify the mineralization of amniotic cells in intact osteodifferentiated hAM and derived amniotic cells (Gualdi et al., 2019). Here, it was reported that, in osteogenic conditions, hAEC had a mesenchymal phenotype with osteocyte function, and even native synthesis of hydroxyapatite, focusing osteogenic potential mainly in this epithelial layer. The whole hAM is characterized using silver nitrate and Alizarin red which are typically used to label the mineralization of isolated cells. Both assays would be easy to implement in a tissue bank.

#### 2.3.2 Functional assays to demonstrate the potential of perinatal derivatives in bone repair

*In vitro*, the ability of fresh and preserved AM to act as a scaffold upon which adult human bone marrow MSC (hBMSC) can proliferate and differentiate into osteogenic lineage has been assessed using metabolic activity assays, alkaline phosphatase staining, and alizarin red quantification (Fenelon et al., 2019; F enelon et al., 2020). *In vivo*, the efficiency of hUC-MSC to promote bone regeneration can be assessed using ectopic or orthotopic models to evaluate their osteoinductive and/or osteoconductive properties. Representative 2D or 3D-radiography followed by histology (Masson’s trichrome and HES staining) are the most commonly performed analysis to evaluate bone regeneration in animal models (Etchebarne et al., 2021; Fenelon et al., 2021). Immunohistochemistry (type I collagen, osteocalcin, AP) brings additional information (Laurent et al., 2017). As an example, it has been investigated the *in vitro* and *in vivo* bone regenerative performance of hUC-MSC and Arginyl-glycyl-aspartic acid (RGD) modified macroporous calcium phosphate cements (CPC). Similarly, it has been observed high expressions of osteogenic specific genes on CPC seeded with hUC-MSC. After implantation for 24 weeks in the rat calvaria bone defect model, the newly formed bone tissue exhibited a higher bone mineral density, new bone amount, and vessel density versus CPC control group (Chen et al., 2013). These observations are in agreement with a study combining blood umbilical cord-derived stem cells with a partially demineralized bone matrix (Liu et al., 2010). Calcium phosphate synthetic bone substitutes (Barboni et al., 2013) and magnesium-enriched hydroxyapatite (MgHA)/collagen-based scaffold (Riccio et al., 2012) were also combined with ovine amniotic fluid PnD. Implanted in an ovine maxillary sinus augmentation model, this study demonstrated a reduced fibrotic reaction, a limited inflammatory response, and an accelerated process of angiogenesis for Sinus explants derived from the PnD-scaffold group compared with the control group (without PnD) (Riccio et al., 2012; Barboni et al., 2013).

Bone develops through a tightly regulated process leading to a hierarchically ordered three-dimensional structure, described in the literature as a bone nodule (Mechiche Alami et al., 2016). UC-WJ-MSC are able to form these 3D structures *in vitro* (Rammal et al., 2017). However, the nodule density of around 9 ± 2 nodules in the cultured area suggests a low commitment of UC-WJ-MSC into osteoprogenitor cells (Rammal et al., 2019). These observations are consistent with other studies highlighting a very low yield of UC-WJ-MSC able to differentiate into osteoblastic lineage (Jin and Lee, 2018; Golchin and Farahany, 2019). Once injected into damaged tissue, UC-WJ-MSC showed a relatively poor rate of cell engraftment and engrafted ones are rather to be short-lived (Wang et al., 2014). The low rate of cell differentiation is in favor of a paracrine mechanism. Despite the small fraction of committed cells, the osteoinductive (i.e., able to induce cell differentiation without adding chemical inductors) scaffold decreased the production of IL-1β, IL-6, and IL-8 inflammatory mediators and increased the release of b-FGF, VEGF angiogenic growth factors by cells in comparison with the inert scaffold. Furthermore, this paracrine action of PnD’s cells enhanced the migration of endothelial cells, the neutrophil diapedesis via ICAM1 up-regulation and the osteogenic potential of endothelial cells (e.g. overproduction of BMP-2) (Rammal et al., 2019).

#### 2.3.3 Clinical assays to demonstrate the influence of PnD on bone repair

Usually, in the clinic, bone union and failure were assessed every 6 months during the first year as follows: first X-ray (or Computed Tomography (CT) scan if doubt) showing 3 of 4 continuous cortices (united) (Moris et al., 2016). Clinically, healing is assessed by the absence of pain and absence of mobility at the fracture site and, in the case of the lower limb, weight-bearing (Gindraux et al., 2020). Some patients could also benefit from the Position–Emission Tomography. In bone repair, osseous biopsies for histological or immuno-histochemistry analysis are often too invasive and usually not performed. When used for example to evaluate the benefit of a specific treatment, markers are comparable to those used *in vivo* (Etchebarne et al., 2021).

#### 2.3.4 Conclusion

In conclusion, for bone regenerative approaches, PnD have been considered in different formats. Although PnD may contribute to bone formation directly, their major value may rely rather on their paracrine activity. The amniotic membrane may act as a collagenous niche for tissue deposition and ingrowth. In consequence, *in vitro* functional assays identifying activities such as immunomodulation, proangiogenic and trophic factor release may represent suitable surrogates for these properties.

### 2.4 Potential role of perinatal derivatives in cardioprotection

Cardiovascular disease and heart failure represent major social and economic burdens as being the leading cause of mortality and morbidity worldwide, with ischemic myocardial infarction the most common event underlying the development of cardiac dysfunction and heart failure (Segers and Lee, 2008; Roger, 2013). When facing prolonged ischemia, the adult mammalian heart can only activate a defective repair mechanism aiming at replacing cardiomyocytes lost during the hypoxic injury with a non-contractile fibrotic collagen scar, leading over time to the detrimental remodeling of the left ventricle, with progression toward myocardial dysfunction and heart failure (Madonna et al., 2016). Despite the remarkable progress achieved by pharmacological and interventional cardiology in the last 20 years, heart transplantation still represents the ultimate therapeutic option for heart failure. Therefore, preclinical research in cardiac regenerative medicine has been recently developed to define innovative therapeutic strategies to enhance cardiac repair mechanisms.

#### 2.4.1 Emerging regenerative approaches for heart repair

*Bona fide* myocardial regeneration can only be obtained by structural and functional reconstitution of compromised/lost cardiomyocytes following injury, via the active renewal of surviving resident ones (Eschenhagen et al., 2017). Such regenerative mechanisms have been extensively described in lower vertebrates. Notably, the neonatal rodent heart has recently been shown to promote proficient myocardial renewal as well, although this response is strictly limited to the very first week after birth, with a clear transition to fibrosis and defective repair soon afterward (Foglia and Poss, 2016). True cardiac regeneration and active myocardial renewal are thus very challenging to be accomplished in the mammalian adult heart; therefore, increasing efforts have recently been focused on optimizing endogenous mechanisms of cardiac repair. These directions focus on enhancing cardio-protection and supporting local angiogenesis, in order to preserve as much viable myocardium as possible during injury/disease. In such a perspective, high expectations have been allocated to paracrine effects from stem/progenitor cells and MSC for cardiac repair (Gnecchi et al., 2012; Hodgkinson et al., 2016; Bollini et al., 2018; Menasché, 2018; Balbi et al., 2020; Magh in et al., 2020). Indeed, the secretome isolated from MSC of different PnD origin has widely been investigated in preclinical cell-free strategies for implementing endogenous myocardial repair as tentative proof-of-principle for future therapy. As a matter of fact, endogenous cardiac repair should be promptly targeted during the early phase following acute heart injury (i.e., following myocardial infarction, at time of reperfusion) or improved by additional therapy in case of off-target cardiotoxic effects due to chronic pharmacological treatment (i.e., chemotherapy).

#### 2.4.2 Functional assays to validate perinatal derivatives in cardioprotection/repair

*In vitro* cardioprotective and pro-angiogenic assays are broadly used to define the paracrine effects of stem/progenitor cell secretomes on supporting cardiomyocyte survival and triggering new vessel development. The *in vitro* mimicking of the altered/injured myocardial conditions can be obtained by exposing cardiomyocytes to severe stress/damage to assess their apoptosis and necrosis, as it has been previously and comprehensively shown (Lazzarini et al., 2016; Balbi et al., 2019a; Balbi et al., 2019b; Takov et al., 2020).

Cardioprotection is usually assessed by evaluating the level of intracellular ROS and metabolism impairment, along with activation of apoptotic pathways and DNA fragmentation in target cardiac cells undergoing hypoxic and/or oxidative stress insult/injury. Cardiomyocyte cell viability is usually evaluated by annexin V and propidium iodide (AnnV/PI) staining, and by MTT/MTS and lactate dehydrogenase activity assay, thus measuring metabolic activity. Angiogenic analyses are generally based on evaluating endothelial progenitor or endothelial-like cell migration and capacity to form tube structures and capillary configurations on Matrigel, following specific stimuli.

The cardio-active and pro-vasculogenic potential of different secretome formulations (the total cell-conditioned medium or its separated and concentrated EV subfraction) from human PnD (i.e., AM-derived progenitors, amniotic fluid stem cells, and umbilical cord stromal mesenchymal cells) have been intensely scrutinized in the last years. The human amniotic fluid progenitor cell secretome and EVs separated and concentrated from that have been recently described to counteract cell death and premature senescence in rodent cardiomyocytes exposed to severe hypoxic stress and cardiotoxic exposure to doxorubicin *in vitro*; likewise, human endothelial colony-forming cells (ECFC) and human umbilical vein endothelial cells (HUVEC) have been shown to respond to the secretome paracrine stimulation by improving their migratory and formation of capillary-like structure *in vitro* (Lazzarini et al., 2016; Balbi et al., 2019a; Balbi et al., 2019b; Takov et al., 2020). Similar promising cardioprotective results have also been obtained with a human amniotic membrane MSC-conditioned medium on the rat embryonic cardiomyoblast-like H9c2 cell line in a model of hypoxia and reoxygenation injury *in vitro* (Danieli et al., 2015). The therapeutic paracrine relevance of human PnD has further been reported in a study demonstrating that human amniotic membrane proteins (AMPs) extracted from the amniotic membrane can antagonize doxorubicin-elicited detrimental effects on H9c2 via the inhibition of oxidative stress and apoptosis. The whole secretome of independent formulation of human umbilical cord mesenchymal stromal cells (UC-MSC), and the EVs obtained from it, has been also proven to be effective in preserving rodent cardiomyocyte cell lines from apoptosis, while stimulating the pro-angiogenic potential HUVEC (Santos Nascimento et al., 2014; Huang et al., 2020).

#### 2.4.3 Conclusion

The majority of studies, reporting *in vivo* validation of PnD therapeutic efficacy (by assessing cardiac function via ultrasound analysis in preclinical animal models of myocardial injury, as extensively reviewed and discussed elsewhere (Magh in et al., 2020)), are to a certain degree supplemented with *in vitro* data describing cardioprotective effects. Some mechanistic studies have been reported, showing as human amniotic fluid stem cell secretome may preserve doxorubicin-stressed murine primary neonatal cardiomyocytes by activating the PI3K/Akt signaling cascade, and by upregulating the NF-κB controlled genes, Il6 and Cxcl1 (Lazzarini et al., 2016). Moreover, human umbilical cord MSC-EVs have been shown to exert beneficial effects as mediating the horizontal transfer of miR-19a, targeting Sox6 in hypoxic H9c2 cells *in vitro*; such candidate mechanism of action was further assessed by means of Sox6 knockdown together with miR-19a inhibition within MSC-EVs (Huang et al., 2020). In conclusion, while most of the studies published so far have reported beneficial effects by using different PnD, consensus on a uniform method for standardized dosing and protocol of administration has not been reached yet.

### 2.5 Potential role of perinatal derivatives in bowel inflammation/intestinal regeneration

Inflammatory Bowel Diseases (IBD) such as Ulcerative Colitis and Crohn’s Disease are chronic inflammatory diseases of unknown etiology. The majority of patients are diagnosed early in life and the incidence is increasing; therefore, the effect of IBD on healthcare systems will rise exponentially.

In the last decade, numerous studies demonstrated as perinatal cells can modulate the activity of inflammatory cells such as macrophages, T and B lymphocytes. The easy cell availability and lack of ethical concerns yield PnD a promising tool in regenerative medicine (Zhang et al., 2017a).

The factors involved in the immunomodulatory actions of perinatal cells have not been fully identified but have been suggested to rise from factors secreted either as soluble proteins (secretome) or entrapped in EVs. Perinatal-derived EVs contribute to modulating the immune response in inflammatory bowel diseases (IBD) by directly targeting different elements of the inflammatory microenvironment, ultimately leading to the repair and regeneration of damaged tissues (Tolomeo et al., 2021).

### 2.5.1 Functional assays to support perinatal derivatives in modulating inflammatory bowel diseases

The efficiency of MSC derived from PnD as well as their secretome (conditioned media or purified EVs) have been tested in chemically-induced experimental colitis mouse models, suggesting as a new and innovative approach for IBD cell-based therapy.

Initial studies have shown that UC-MSC modulate dextran sulfate sodium (DSS) induced acute colitis in NOD.CB17-Prkdc scid/J immunodeficient mice (Banerjee et al., 2015). Recently, human UC-MSC effectively alleviated weight loss, intestinal mucosal injury, colon shortening, and reduced clinical disease phenotype in the DSS-induced mouse IBD model by inhibiting ERK phosphorylation in neutrophils (Wang et al., 2020). In a similar experimental approach, both efficacy and mechanism of action of UC-MSC were further evaluated in acute and chronic mouse models of IBD, established by using a DSS solution. Findings of this study indicated an enhanced expression of the intestinal tight junction protein occludin, downregulation of the expression of the autophagy marker LC3A/B in colon tissue, and upregulated the expression of VEGF-A and VEGFR-1 at the injured site (Panhua et al., 2019). In addition, in a DSS-induced colitis model UC-MSC, when administered with MIS416, a novel microparticle that activates NOD2 and TLR9 signaling was found to improve the pathological phenotype by systemically regulating the immune response, (Lee et al., 2018). In this context, treatment with hAFSC resulted in preventing colon shortening after induction of colitis and dramatically ameliorated the body weight loss induced by the DSS treatment

Similar results were obtained when UC-MSC were administered in a trinitrobenzene sulfonate (TNBS)-induced mouse model of colitis (Liang et al., 2011). More specifically, the administration of UC-MSC reduced the severity of TNBS-induced colitis in mice by increasing anti-inflammatory responses in colons of mice, such as the production of IL-10 and reduced production of inflammatory cytokines (Kim et al., 2013). Moreover, preconditioning of UC-MSC with polyribocytidylic acid enhanced the therapeutic effects of UC-MSC in TNBS-induced colitis (Qiu et al., 2017).

Notably, a number of studies showed as AF-MSC and UC-MSC-derived secreted molecules had a significant therapeutic effect, suggesting a paracrine anti-inflammatory mode of action. Indeed, secretome studies on AF-MSC indicated the presence of anti-inflammatory molecules such as interleukins IL-10, IL-1ra, IL-13, and IL-27; and angiogenic factors like angiopoietin-1, PD-ECGF, uPA that can contribute to the functional improvement of DSS-induced colitis in immunodeficient colitis mouse model phenotype (Legaki et al., 2016). Similarly, intraperitoneal injection of MSC extracts in the DSS-C57BL/6 mouse model reduced colitis, disease activity index, histological colitis scores, and increased body weight (Song et al., 2017). EV-based strategies, including UC-MSC-EVs exerted profound effects on alleviating DSS-induced IBD through the modulation of IL-7 expression in macrophages (Mao et al., 2017b) and induction of MUC2 expression (Tolomeo et al., 2021). Furthermore, recent studies indicated that UC-MSC-EV samples containing miRNA-378a inhibited NLR family pyrin domain containing 3 (NLRP3) inflammasomes and abrogated cell pyroptosis, (Cai et al., 2021) and also restored mucosal barrier repair and intestinal immune homeostasis via TNF-α stimulated gene 6 (Tsg6), to protect against DSS-induced colitis (Yang et al., 2021).

### 2.5.2 Functional assays to demonstrate the influence of perinatal derivatives in clinic for inflammatory bowel diseases

Experimental approaches have been translated into clinical settings by using stem cell-based therapies for IBDs. Recent data showed that local administration of autologous MSC into perianal fistula leads to an enhanced healing process, promoted fistula relief, and ameliorated clinical complications (Hossein-khannazer et al., 2021).

Interestingly, administration of perinatal MSC to patients with Crohn’s disease was proved safe and well-tolerated, generating decreased Crohn’s disease activity index score and complete remission. Similarly, the application of allogeneic UC-MSC exhibited safety, feasibility, limited or no adverse effects, and led to the improvement of clinical symptoms. However, no significant changes in the levels of inflammatory cytokines in blood serum were detected (Hossein-khannazer et al., 2021; Vieujean et al., 2022).

### 2.5.3 Conclusion

Overall, cell-based therapy in IBDs demonstrated that MSC are safe and beneficial following anti-inflammatory activity. However, cell-free products (EVs or secretomes in general) may represent an ideal source of anti-inflammatory agents, solving the problem of cell safety and toxicity (Villa et al., 2019; Herrmann et al., 2021). Notwithstanding, additional studies are ongoing to optimize and standardize preparation, administration, and dosage. Accordingly, the development of functional assays to validate PnD products is a prospective direction to modulate microbial structure or intestinal homeostasis.

### 2.6 Potential role of perinatal derivatives in liver regeneration

Liver dysfunction comprises heterogeneous malfunctions that could potentially lead to acute or chronic liver disease, such as fulminant hepatitis or acute liver disease, advanced fibrosis/cirrhosis, congenital metabolic disorders, or fatty liver disease (Psaraki et al., 2021). Liver transplantation is the gold-standard therapy for many of the afore disorders, limited by organ shortage and the need for a lifelong immunosuppressive regimen. Recent refinements and preclinical/clinical studies have validated alternative approaches where fetal or neonatal liver cells have been infused in patients with chronic or congenital liver disorders (Gramignoli et al., 2015; Bluhme et al., 2022).

Despite innate proliferative capacities, these progenitor cells have shown limited metabolic and synthetic strength, with reduced clinical outcomes. Furthermore, the limited availability of adult human hepatocytes for clinical treatments, in addition to the immunosuppressant regiment required to sustain cellular long-term engraftment have encouraged the use of different cell sources. Over the past years, several groups and companies have been proposing stem cells as an alternative solution, bounded by genetic and epigenetic instabilities, and limited by hepatic maturation level (Bluhme et al., 2022). The additional negative caveat is represented by the chronic inflammatory milieu in response to allograft, which may favor the oncogenic transformation of transplanted cells. Immune-privileged cells such as perinatal stem cells have attracted attention in preclinical studies. Cells isolated from AM have satisfied safety and efficacy requirements, overcoming all the aforementioned risks, and reverting life-threatening acute and congenital liver diseases. Encouraged by lack of tumorigenicity and multipotency potential, hAEC have been transplanted and corrected inborn errors of metabolism such as maple syrup urine disease (MSUD) (Skvorak et al., 2013a; Skvorak et al., 2013b). Phenylketonuria (Strom et al., 2013), and another liver-based disease, is associated with end-stage renal pathologies (atypical hemolytic uraemic syndrome) (Casiraghi et al., 2021). Preclinical analysis revealed correction or normalization in amino acid and neurotransmitter imbalances in human perinatal treated animals, to a superior level reached by syngeneic murine hepatocytes. And the lack of immuno-recognition in xenogenic settings raised attention to the immune-evasive capacity amniotic cells have, rather than an immuno-privileged phenotype.

Allogeneic perinatal cells have been infused to rescue livers in acute failure, supporting parenchymal cell survival and hepatic functions, highlighting remarkable engraftment and anti-inflammatory effects. Both MSC isolated from amniotic fluid and chorionic plate have proved their potential in rescuing intoxicated livers (Lee et al., 2010; Zagoura et al., 2012; Peng et al., 2014).

Xenotransplantation of perinatal cells into immune-competent animals has proved long-term acceptance without the administration of immunosuppressive drugs and resulted in hepatoprotection and regulation of the inflammatory process (Milosavljevic et al., 2017; Shi et al., 2017). However, in clinical situations, liver treatments by allogeneic cells have been proved to require immediate availability (in fulminant hepatitis) and a high number of cells (up to 2 × 10^8^ cells per kg of body weight in metabolic disorders) (Gramignoli et al., 2015). Such challenges have been frequently hampered by limited engraftment capacity by donor cells, as well as lack of selective advantage. Recent clinical trials have tested the supportive role offered by UC-MSC to allogeneic human hepatocytes for the treatment of liver disease (Iansante et al., 2018). Different reports highlighted as injection of UC-WJ-MSC in patients with chronic liver disorders or steatohepatitis ameliorate transaminase levels and lipid profile, reduces bilirubin and hyperglycemic profile, resulting in improved MELD score and overall survival (see (Shahrbaf et al., 2022) for revision of such clinical studies). Beneficial clinical support offered by perinatal cells has been ascribed to repeated infusion rather than one single infusion (Jia et al., 2020), in line with results offered by 3 decades of hepatocyte transplantation (Gramignoli et al., 2015). Another source of perinatal MSC, amniotic membrane, has been described as instrumental to attenuate Kupffer autophagy and prevent Stellate activation (Shahrbaf et al., 2022). Recently, it has been reported the therapeutic effects of intact AMSC and secreted factors in reversing sclerosing cholangitis (Sugiura et al., 2018). The pro-regenerative effect offered by perinatal MSC in advanced fibrotic/cirrhotic conditions has been ascribed to ECM remodeling factors, such as secreted MMPs (Fu et al., 2018), or angiogenic and hepatogenic mediators (Ding et al., 2018; Hoffmann et al., 2020). Indeed, perinatal cells actively interact and crosstalk with (innate and adaptive) immune cells or support hepatic regeneration not only by cell-to-cell interactions but also through paracrine mediators. Several studies suggest as perinatal therapies, adequate for patients with liver diseases exacerbated by deranged inflammation, will most likely benefit from PnD secretome rather than cellular hepatic maturation (Nagaishi et al., 2014; Liu et al., 2015; Trohatou and Ro ubelakis, 2017). The clinical efficacy of CP-MSC or AF-MSC injections has been described in relation to secreted anti-inflammatory (i.e., TGF-β1 and IL-10) or anti-fibrotic (i.e., MMP-2 and MMP-9) mediators (Shahrbaf et al., 2022). But soluble mediators are characterized by shorter half-life and limited paracrine distribution compared to active molecules (surface-bound or miRNA) embedded in EVs.

#### 2.6.1 Hepato-specific functional assays to validate perinatal derivatives in liver disease

In the majority of the published reports, the cell viability test has been the sole evaluation for donor quality before infusion. Viability quantification has been routinely offered and used to validate cell transplantation procedures. Regulatory Agencies, such as FDA or EMA, mandate parameters such as product sterility, cell viability, and identity to be included to validate cellular biopharmaceutical products. PnD products are not relieved by such qualifications. While product sterility (bacteria- or mycoplasma-free) is quite a standardized procedure, routinely performed in every manufacturing site, currently, no consensus on the level of viability for the cell product is widely accepted. In many phase II trials, MSC viability between 70% and 90% is commonly required (Matthay et al., 2019), while primary liver cell preparation cannot be inferior to 60% in viability to allow injection in patients (Gramignoli et al., 2015). Furthermore, cell viability is seldom performed to determine the percentage of viable cells versus death (necrotic) cells, with additional detection of cells in the early stages of the cell death cascade (i.e., apoptosis).

The International committee described epithelial cells isolated from the fetal side of the amniotic membrane as positive for CD49f and CD326 (ISCT committee; (Dominici et al., 2006)) and published a position paper recommending the inclusion of functional assays to couple with identity validation for therapeutic cell products (Viswanathan et al., 2019). Nevertheless, current biosynthetic analysis or quantification of secreted anti-inflammatory/anti-fibrotic mediators can be instrumental only for a therapeutic approach where no stem cell maturation/transdifferentiation is required. Hepato-specific phase I and II activities, bile acid transporters, and urea cycle enzymes are critical to determining long/term correction of congenital liver disorders. In hepatocyte transplant programs, such a multiparametric functional analysis is commonly the minimum criteria in order to confirm cell function (Donato et al., 2008; Bluhme et al., 2022).

Between perinatal cells, hAEC have attracted particular attention for their hepatocyte-like features and can acquire hepatic functions upon transplantation. Epithelial cells released by the amniotic membrane have been proved to secrete low levels of albumin, and upon engraftment in liver parenchyma mature into functional hepatocyte-like cells expressing Cytochrome P450 (CYP) enzymes (as CYP3A4) or secreting proteinases (as A1AT) (Takashima et al., 2004). *Ex vivo* maturation of stem or progenitor cells into a functional hepatic phenotype is still debated. During the past years, revisional studies elegantly proved as the generation of hepatocyte-like cells starting from somatic or perinatal MSC was not a direct consequence of trans-differentiation into endoderm-like cells, but rather resulting from the fusion between donor cells and recipient’s hepatocytes (Camargo et al., 2004; Willenbring et al., 2004). Differently, epithelial cells isolated from the amniotic membrane have been proved capable to mature into functional, adult parenchymal cells upon being implanted in the liver, precluding any fusion event between donor and recipient cells (Marongiu et al., 2015). Such hepatic maturation has only been partially achieved in 2D culture (M iki et al., 2005; Marongiu et al., 2011; Fanti et al., 2017; Maymó et al., 2018; Passaretta et al., 2020).

Metabolic activities in human hepatocytes or stem cell-derived hepatocyte-like cells can be assessed by incubation with drugs or chemical substrates and analyzed with unexpensive and widely available lab equipment (such as spectrophotometers) (Kim et al., 2005; Gramignoli et al., 2012). Release criteria and a battery of potency assays have been developed and described, where up to eleven different hepatic functions can be simultaneously measured to validate donor cells transplanted to offer support or replace liver cells (Gramignoli et al., 2013; Gramignoli et al., 2014). Indeed, in order to validate multi-functional cells, such as liver cells, a matrix of functional assays has been recommended to support injection of multipotent PnD cells and confirm maturation capacity into periportal or centro-lobular hepatocytes and cholangiocytes. Bio-synthetic and metabolic activities have been frequently coupled with selective transcriptome analysis. Hepatic maturation can be monitored by transcriptomic assays specifically designed to validate stem/progenitor cell transplantations and match patients’ needs (Zabulica et al., 2019).

#### 2.6.2 Liver organoid as novel perinatal derivatives maturation potency assay for liver disease

The therapeutic potential of liver organoids represents an alternative of functional and expandable cells for transplantation, exceeding the current limitations (Prior et al., 2019). Liver organoids have been recently established from different cell sources among which hAEC have currently gained particular attention.

Some authors demonstrated that the combination of different cell types (endothelial, mesenchymal) in a 3D culture system may promote and enhance stem cell differentiation, physiological function, and proliferation ability, providing a tissue engineering strategy, referred to as “building blocks”, for larger tissue constructs (Takebe et al., 2013; Laschke and Menger, 2017; Nie et al., 2018; Lebreton et al., 2019). Recently, multicellular organoids have been generated with hepatic features and functions by combining hAEC with HUVEC and MSC (Furuya et al., 2019). Besides obtaining functional organoids, they evaluated cellular differentiation within organoids through a bioinformatics approach demonstrating a higher level of cell differentiation in comparison with 2D cell cultures.

Additional studies have been conducted to evaluate liver organoids for hepato-specific gene expression and functions, such as CYP3A4 activity and inducibility, albumin secretion, ammonia and metabolism, the ability to efflux rhodamine or store bile acids, lipids, or glycogen (Ramachandran et al., 2015; Pettinato et al., 2019; Wu et al., 2019).

Based on these results, liver organoids could represent a novel *in vitro* model to study novel therapeutic interventions, but may become important an alternative strategy to organ transplantation for liver failure (Furuya et al., 2019). However, implantation strategies and high engraftment efficiency are crucial to rescue metabolic defects. The percentage of organoid engraftment reported is extremely low (1%) (Huch et al., 2013) and restoring enzyme or protein deficiency requires several billion proficient cells, quantified in approx. 5–10% of total liver mass according to missing enzyme (Gramignoli et al., 2015). It has been reported as liver organoids can mimic structures and genetic signatures, although their biomedical use on a large scale is currently limited to the lack in control of cell size, shape, and composition (Rossi et al., 2018). In this regard, it appears crucial to optimize long-term cultures and differentiation characteristics of liver organoids aimed at generating a tissue architecture closer to the *in vivo* conditions (Ramachandran et al., 2015). *In vitro* refinements as well as *in vivo* validation are in progress.

#### 2.6.3 Conclusion

The liver, with its unique regenerative capacity and plethora of functions critical for life, is most likely one of the organs that can largely benefit from PnD supportive strategies to enhance regeneration rather than organ replacement.

Cell-based therapies have proved safety and efficacy for liver regeneration or inborn errors of metabolism correction. However, a reliable, stable source of functional hepatocyte-like cells is still pending to offer such a strategy to millions of patients with congenital or chronic liver disease. Perinatal cells such as MSC or hAEC have been recognized with constitutive and robust anti-inflammatory and anti-fibrotic properties, qualifying these PnD as suitable bioproducts for inflammatory conditions such as hepatitis, fibrosis, and cirrhosis. However, to correct enzymatic defects, a large amount of proficient and metabolic active cells is needed. Convincing evidence in support of the maturation of epithelial cells isolated from AM is encouraging. Moving from the fact that hAEC originates from epiblast during the second week of embryo development and display the presence of early-phase hepatic markers, such PnD product is motivating translation into phase I and II clinical trials. Conversely, transdifferentiation of MSC into functional hepatocyte-like cells has raised more concerns than solid evidence and will most likely restrict their use to paracrine effects or secretome approaches in the near future.

Major challenge in many published studies is validating progenitor/stem cell maturation into functional hepatocytes. Transcriptomic analysis has represented an easy and relatively cheap method to initially test PnD, but such analysis needs to be coupled with real metabolic activity and performed in direct comparison with primary human hepatocytes.

The involvement of metabolic enzymes characteristic of mature, functional somatic cells and the current lack of standardized and reproducible protocols able to induce hepatic maturation on any progenitor or stem cell *in vitro* to a level that corresponds to postnatal liver cells (Zabulica et al., 2019) makes hepatic potency assay refinement even more complex than any other organ, where immunomodulation or inflammatory governance is the sole or major goals.

Finally, concerning the liver organoid approach, whilst many 3D liver models are described as organoids, not all of them are equally organotypic since in most cases they are aggregates of a single cell type or mixed cell types rather than containing both parenchymal and non-parenchymal cells of the liver necessary for modeling inflammation and liver disease. Although this approach still needs further validation *in vitro* and *in vivo*, to circumvent the issue of angiogenesis, innervation or survival of encapsulated hepatocytes, PnD-derived organoids deserve much attention in comparison both with iPSC-derived organoids, which imply the risk to develop teratomas and with adult stem cells-derived organoids which show a lack of heterogeneity in the starting population.

### 2.7 Potential role of perinatal derivatives on lung fibrosis

Idiopathic pulmonary fibrosis (IPF) is a progressive, fatal, chronic interstitial lung disease characterized by aberrant extracellular matrix deposition leading to loss of normal lung architecture and respiratory capacity. In Europe and North America, IPF has an incidence of 3–9 cases per 100,000 people and is increasing worldwide (Scelfo et al., 2017).

Although IPF etiology remains unknown, it is generally considered a dysregulated wound healing process mediated by resident fibroblasts and recruited fibrocytes which are activated to myofibroblasts (the latter of which are mostly responsible for extracellular matrix production) by repetitive alveolar injuries (induced for example by environmental exposures, smoking, viral infections and by genetic predispositions). However, more recent findings evidenced the contribution of diverse immune cell types, belonging to both innate (neutrophils, monocytes, macrophages) and adaptative (T and B lymphocytes) immune systems, in the initiation and progression of fibrotic lesion in IPF patients (Desai et al., 2018; Heukels et al., 2019).

In this context, PnD have been applied in lung fibrosis models with the purpose either to counteract the proliferation and collagen production of lung myofibroblasts and also to modulate the inflammatory response involved in fibrosis initiation and progression.

#### 2.7.1 Lung-specific functional assays to validate perinatal derivatives in lung fibrosis

Due to the multiple cellular actors involved in the pathogenesis of IPF and the consequent implication of different pathways contributing either to fibrogenesis and fibrolysis, *in vitro* models of fibrosis do not resemble the complex conditions of *in vivo* fibrotic tissues. The general *in vitro* approach is to analyze the effects of PnD on *in vitro* models of inflammation-independent fibrosis, for example, the assessment of their direct effects on the proliferation, collagen production, and the secretion of profibrotic cytokines (e.g., TGF-β, PDGF-a, and PDGF-b), of lung fibroblasts activated to myofibroblasts with TGF-β (Li et al., 2008; Vosdoganes et al., 2013).

Recently, 3D *in vitro* lung models have been developed in the effort to reproduce the cellular and functional complexity of pulmonary tissue and possibly recapitulate the cellular and structural alterations present in the lungs of patients with IPF. Lung organoids have been developed by combining collagen-functionalized alginate beads and induced pluripotent stem cell (iPSC) derived from human lung fibroblasts in a rotational bioreactor (Wilkinson et al., 2017). To generate a model of progressive scarring, as found in IPF patients, organoids have been treated with exogenous TGF-β1 and observed increased expression of collagen 1, α-SMA, and the emergence of fibroblastic foci confirming the possibility to model lung fibrosis *in vitro*.

Lung spheroids (called pulmospheres) have been developed using cells and extracellular matrix components isolated from lung biopsies obtained from IPF patients (Surolia et al., 2017) and they have been applied to evaluate the responsiveness of individual patients to antifibrotic drugs.

The limitations of the *in vitro* lung disease modeling make it so that studies focused on establishing the potential anti-fibrotic effects of PnD have been performed on animal models evaluating a series of cellular/humoral/tissue parameters. Specifically, PnDs, and mainly hAEC and hAMSC, have been administered in animals with bleomycin-induced pulmonary fibrosis to evaluate either their ability to reduce lung inflammation and their potential to slow down the progression and even to reverse the fibrotic lesion.

In these studies, the anti-inflammatory ability of PnD was evaluated in terms of reduction of inflammation and immune cell infiltration in lung parenchyma and broncho-alveolar lavage. Specifically, these were observed as: 1) a reduction of neutrophils (Cargnoni et al., 2009; Murphy et al., 2011) and macrophages in the lungs (Tan et al., 2014); 2) increased lung levels of regulatory T cells (Treg), able to suppress T cell activation (Tan et al., 2015); 3) promotion of macrophage polarization toward an anti-inflammatory phenotype (M2 macrophages) (Murphy et al., 2012; Tan et al., 2014; Tan et al., 2018); 4) reduction of lung levels of proinflammatory cytokines (MCP-1, TNF-a, IL-1, IL-6) (Moodley et al., 2010; Murphy et al., 2011) and chemokines (CXCL12, CXCL13, BAFF (Carg noni et al., 2020)); 5) increase of tissue levels of cytokines and molecules with anti-inflammatory activities, such as IL-10 (Carg noni et al., 2020) and TSG-6 (Moodley et al., 2013); 6) reduction in the number and in the maturation ability of B cells recruited in lung parenchyma (Carg noni et al., 2020); and 7) reduction in the number and size of Tertiary Lymphoid Organs (TLO) (Carg noni et al., 2020), T and B cell aggregates found in the lungs of IPF patients and that correlate with inflammation chronicization and severity of lung fibrotic lesions (Todd et al., 2013).

Associated and possibly mediated by the ability to counteract the inflammatory response and the chronicization of lung inflammatory processes, PnD (hAEC and hAMSC) exhibited the ability to interfere with the progression of lung fibrotic lesions. Lung fibrosis has been evaluated through the assessment of parameters/factors involved either in fibrogenesis or in fibrolysis. Indeed, it has been observed that PnD decreases lung levels of TGF-β1 (Moodley et al., 2010), a pro-fibrotic growth factor with a crucial role in triggering the activation of lung fibroblasts to myofibroblasts (α-SMA positive cells) (Murphy et al., 2012; Vosdoganes et al., 2013; Carg noni et al., 2020) and lung accumulation of collagen and fibronectin (Vosdoganes et al., 2013; Carg noni et al., 2020). Instead, PnD appears to promote fibrolysis by increasing lung levels of MMPs and by decreasing their inhibitors TIMPs (Moodley et al., 2010).

UC-MSC, similar to hAEC and hAMSC, were recently reported to reduce collagen deposition and lung IL-6 expression in bleomycin-challenged mice when administered twice (24 h and 7 days post-bleomycin) (Moroncini et al., 2018). However, differently from the other PnD, hUC-MSC treatment reduced lung levels of M2 macrophages.

It must be underlined that the anti-inflammatory and anti-fibrotic effects of perinatal cells were observed regardless of any significant engraftment of these cells in the injured lung after administration (Cargnoni et al., 2009; Murphy et al., 2011), suggesting that secreted factors, rather than cells per sé, act via a paracrine mechanism on host cells. Indeed, both conditioned medium generated from the *in vitro* culture of hAMSC (hAMSC-CM) and EVs from hAEC (hAEC-EVs), have been shown to exert beneficial actions in a bleomycin-induced lung fibrosis model. The treatment with hAMSC-CM preserved blood gas parameters of bleomycin-challenged mice and reduced lung levels of pro-inflammatory and pro-fibrotic cytokines (IL-6, TNF-α, MIP-1α, MCP-1 TGF-β) associated with reduced lung macrophage levels (Cargnoni et al., 2014). Intranasal instillation of hAEC-Exo administered early, on day 1 post bleomycin challenge, reduced lung inflammation while treatment on day 7 improved tissue-to-airspace ratio and reduced fibrosis (Tan et al., 2018).

#### 2.7.2 Conclusion

The experimental evidence supporting the anti-inflammatory and anti-fibrotic actions of PnD (cells and derived secretome and EV) has encouraged their application in clinical studies. In particular, placental stromal cells (Chambers et al., 2014) and hUC-MSC (Zhang et al., 2017b) were transplanted in IPF patients with null/mild side effects. More recently, to limit the immunopathogenic effects of cytokine storm, hUC-MSC were used, in combination with the standard optimal care, to treat COVID-19 patients with ARDS (Meng et al., 2020; Shu et al., 2020). The studies performed until now indicated that PnD administration is feasible and safe (Lanzoni et al., 2021), however, clinical trials with a large population of patients are needed to demonstrate the potential efficacy of PnD in human diseases.

## 3 Overall message

The use of PnD in regenerative medicine covers the employment of scaffolds after decellularization or de-epithelization, stem cells, and secretome including EVs. Recent preclinical and clinical results propose PnD derivatives as safe and effective biological products, whereas their therapeutic application and effectiveness remain challenged. Potency assays are designed to certify the mechanism of action of starting material, pre- or post-commitment.

Any PnD technology is required to satisfy strict release criteria, based on the quality of cell products as well as potency measurements validating specific capacity. Unlike conventional release criteria based on static marker qualification (immune-cytological analysis), specific potency assays are less standardized and cannot be performed on donor cells inefficiently or partially committed over a specific phenotype.

Multiple factors influence treatment outcomes and the proposed potency assays aim to mark a new path to standardize PnD application. This manuscript aimed at offering a guide on when and how it may be advantageous for the employment of placenta-derived cells or scaffolds. The described potency assays can serve as the first tool to standardize PnD derivation and usage, and the second instance may contribute to match recipients’ needs with donor characteristics. We are aware that standardization in manufacturing and qualification represents the main limitations hampering a more intensive and extensive use of PnD in clinical trials, so far. A current lack of consensus in the scientific community has limited PnD use in clinical settings, although such derivatives have been reported well-tolerated and refractory to the host’s immunorecognition and rejection. To establish and predict PnD functionality, COST working group of experts has been actively recruited and worked to establish a clear set of laboratory tests that can be conducted in every research or transplant centers, using common laboratory equipment and fulfilling regulatory agencies’ requirements. Different validation analyses will likely require both *in vitro* 2D/3D tests to refine activity and modalities, correlated by *in vivo* validations using human-related animal models. As result, we can envision in the near future, a guide compiled by the international community that will standardize the assays, detail advantages and disadvantages, and tailor the implementation of PnD according to patient’s needs. Any ethical or religious issues have been depicted during the past years, since perinatal cells and tissue are commonly considered waste material (at least in the vast majority of medical centers) and may represent a new perspective in regenerative and interventional medicine, upon standardization and validation of the afore processes.

## Data Availability

The original contributions presented in the study are included in the article/Supplementary Material, and further inquiries can be directed to the corresponding authors.

## Glossary

α-SMA: α- smooth muscle actin
AMP: amniotic membrane protein
AMSC: amniotic membrane mesenchymal stromal cells
AnnV/PI: annexin V and propidium iodide
AP: alkaline phosphatase
BBB: blood-brain barrier
bFGF: basic fibroblast growth factor
CD: Crohn’s disease
CM: conditioned medium
CMAP: compound muscle action potential
CNS: central nervous system
CPC: calcium phosphate cements
CT: computed tomography
DAMP: danger-associated molecular patterns
dP: decellularized perinatal derivative
dP-ECM: dP extracellular matrix
DSS: dextran sulfate sodium;
ECFC: endothelial colony-forming cells
EDX: energy dispersive X-ray
EGF: epidermal growth factor
EMG: electromyologram
EPT: extensor postural thrust
ES: embryonic stem cells
EV: extracellular vesicles
GFAP: glial fibrillary acidic protein
GT: grasping test
hACM: human amnio-chorionic membrane
hAEC: amniotic membrane epithelial cells
hAEC-Exo: exosomes from hAEC
hAFSC: amniotic fluid stem cells
hAF-MSC: amniotic fluid mesenchymal stromal cells
hAM: human AM
hAMSC-CM: human amniotic membrane mesenchymal stromal cells conditioned medium
hBMSC: human bone marrow mesenchymal stromal cells
HE: hematoxylin and eosin;
HUVEC: human umbilical vein endothelial cells
IBD: inflammatory bowel diseases
IFNγ: interferon-gamma
IGF: insulin-like growth factor
IL: interleukin
iPSC: induced pluripotent stem cells
IPF: Idiopathic pulmonary fibrosis
LPS: lipopolysaccharide
MgHA: magnesium-enriched hydroxyapatite
MMP: metalloproteinase
MSC: mesenchymal stromal cells
MSCEVs: MSC- derived extracellular vesicles
MSCMVs: MSC-derived microvesicles
MSUD: maple syrup urine disease
MVs: microvesicles
NCV: nerve conduction velocity
NLRP3: NLR family pyrin domain containing 3
NPC: progenitor cells
NSC: neural stem cells
OGD: oxygen-glucose deprivation
OPC: oligodendrocyte progenitor cells
PAMP: pathogen-associated molecular patterns
PCL: polycaprolactone
PDGF: platelet-derived growth factor;
PLA: polylactate
PLGA: poly(lactic-co-glycolic acid)
PnD: perinatal derivatives
PNS: peripheral nervous system
ROS: reactive oxygen species
RGD: Arginyl-glycyl-aspartic acid
SFI: sciatic functional index
TEER: Trans endothelial electric resistance
TGF- β1: transforming growth factor beta 1
TIMP: tissue inhibitor of metalloproteinases
TNBS: trinitrobenzene sulfonate;
TNFα: tumor necrosis factor-alpha
UC-MSC: umbilical cord mesenchymal stromal cells
UC-WJ-MSC: Umbilical cord Wharton’s jelly mesenchymal stromal cells
VEGF: vascular endothelial growth factor
VML: volumetric muscle loss
WRL: withdrawal reflex latency
XRD: X-ray diffraction.
